# Uncertainty-aware joint modeling for Sanskrit compound splitting and segmentation

**DOI:** 10.3389/frai.2026.1873672

**Published:** 2026-07-28

**Authors:** Irfan Ali, Liliana Lo Presti, Marco La Cascia, Igor Spano'

**Affiliations:** 1 Department of Engineering, University of Palermo, Palermo, Italy; 2 Department of Cultures and Society, University of Palermo, Palermo, Italy

**Keywords:** CRF-model, deep learning, low-resource language, Sanskrit language, transformer, word segmentation

## Abstract

Sanskrit compound word splitting is challenging because Sandhi-driven phonological transformations obscure word boundaries, making splitting non-deterministic, especially in multi-split cases where multiple hidden boundaries must be identified and constituent segments reconstructed. In this study, a joint end-to-end Transformer-based multi-task architecture is proposed to address this problem by integrating boundary detection and segmented sequence generation within a unified framework. The model employs a shared character-level Transformer encoder that feeds a BiLSTM-CRF boundary prediction branch, which produces sequence-consistent boundary locations and posterior marginals, and a Transformer decoder that generates the segmented output autoregressively. To couple the two tasks, boundary probabilities and entropy-based uncertainty are derived from the CRF marginals. These signals are used to gate encoder representations and to construct a global boundary-aware context that conditions each decoding step, thereby enabling more robust decoding under boundary ambiguity. Experimental results demonstrate consistent improvements over state-of-the-art methods. The model achieves exact-match boundary location accuracy of 84.39%, exact segmentation accuracy of 79.34%, and character-level accuracy of 87.79%. It also achieves boundary-level Precision, Recall, and F1 scores of 92.84%, 91.66%, and 92.25%, respectively. These results indicate that uncertainty-aware coupling and structured BiLSTM-CRF supervision improve segmentation performance while maintaining strong boundary detection, thereby enabling more accurate morphological analysis for NLP and digital humanities applications.

## 1 Introduction

Sanskrit is an ancient Indo-Aryan language, first written around 1500 BCE (Hellwig, 2015b; Ali et al., 2025b), with a large and culturally significant corpus (Huet, 2003). The grammar was fully codified in Panini's Astadhyayi (Cardona, 1997), a work that continues to influence linguistic theory today.

Computational processing of Sanskrit is challenging due to its rich morphology, free word order, extensive compounding, and the pervasive phonological phenomenon of Sandhi. Despite large-scale digitization efforts such as the Digital Corpus of Sanskrit (DCS) (Hellwig, 2024), the use of Sandhi—i.e., phonological transformations occurring at morpheme boundaries—remains a major source of difficulty in splitting Sanskrit words (Aralikatte et al., 2018).

In Sanskrit, compound words can be divided at several positions, and multi-split Sandhi, in which a single word contains three or more split points, makes segmentation particularly difficult. Indeed, phonological changes such as vowel mixing, consonant merging, and visarga deletion accumulate, increasing uncertainty about the true boundaries.

Additionally, some candidate segmentations may be phonologically well-formed yet semantically improbable, requiring the integration of phonological, lexical, and semantic knowledge to rule out false interpretations. For instance, the short string *h*ā*h*ā*k*ā*r*ā (“the exclamations ‘hāhā”) has 625 splits under the Sandhi rules reported in Hellwig (2015b). The correct disambiguation thus depends on semantic compatibility among candidate segmentations. Tokenization is a key element of NLP but the previously highlighted ambiguity complicates the task.

Consequently, Sandhi splitting, especially in multi-split scenarios, remains challenging because it requires accurate boundary detection and reconstruction of the original units. Nonetheless, it is important because it supports several downstream tasks, such as text classification, morphological tagging, dependency parsing, and automatic speech recognition.

Two common methods for Sanskrit word segmentation are the lexicon-based and data-driven approaches. Lexicon-based methods rely on external linguistic resources, whereas rule-based methods cannot handle exceptions and variations. Data-driven techniques learn segmentation patterns from annotated data and often formulate the problem as a structured prediction or sequence-labeling task. Classic approaches (Dave et al., 2021; Aralikatte et al., 2018) rely on a two-step process: (i) identify the point of split, (ii) estimate the constituent words. Errors in boundary detection lead to false predictions of the constituent words. While attention-based models (Ali et al., 2025a,b) can minimize splitting ambiguity, they are typically limited to single-split compounds and fail to handle multi-split cases. Conversely, Ali et al. (2026) attempts to detect multiple split locations using an iterative threshold-based prediction method, without prior knowledge of their number. However, the method does not generate the segmented outputs. A key limitation of current approaches is the lack of explicit modeling of boundary uncertainty. In Sanskrit Sandhi, boundary decisions are inherently ambiguous and context-dependent, yet most models collapse this uncertainty into point estimates before generation. This leads to error propagation and limits the model's ability to recover correct segmentations when boundary predictions are uncertain or partially incorrect.

In this study, we propose an uncertainty-aware joint modeling framework that integrates structured boundary prediction with conditional sequence generation. Rather than relying on hard boundary decisions, we use posterior marginals from a CRF-based boundary model to derive boundary probabilities and uncertainty estimates. These signals modulate encoder representations and construct a global boundary-aware context that conditions the decoder at every step. This design enables the model to explicitly reason about ambiguous boundary configurations, improving robustness in multi-split scenarios. More broadly, our approach provides a general mechanism for incorporating structured uncertainty into sequence generation models, applicable to other tasks involving latent structure and ambiguous segmentation.

We conduct extensive experiments on multiple benchmark datasets from the literature, covering both single- and multi-split scenarios. We also perform ablation studies to systematically evaluate the contribution of each component of the proposed architecture. Furthermore, we construct a benchmark dataset that includes compound words with multiple split points (up to five splits and 96 characters per word), enabling evaluation under more complex segmentation settings.

Overall, the core contributions are:

- *Joint modeling of boundary detection and segmentation*. We formulate Sanskrit compound splitting as a unified task that jointly models boundary localization and character-level generation within a single architecture, enabling consistent handling of multi-split compounds.
- *Structured boundary modeling with uncertainty-aware signals*. We employ a BiLSTM-CRF module to model dependencies among boundary labels and capture multiple split positions, and further use CRF posterior marginals to derive boundary probabilities and uncertainty estimates.
- *Uncertainty-aware coupling between boundary prediction and generation*. We introduce a learned gating mechanism and a boundary-aware global context that use CRF-derived signals to modulate encoder representations and condition the decoder, enabling robust segmentation in the presence of boundary ambiguity.
- *Multi-split benchmark dataset*. We construct a benchmark dataset for Sanskrit compound splitting that includes more than 89*k* compound words of varying complexity, with up to 96 characters per word and up to five split locations.

## 2 Related work

In this section, we review prior work on word segmentation and Sanskrit Sandhi splitting, focusing on approaches to boundary modeling and sequence generation, as well as the limitations of these approaches in capturing uncertainty in multi-split scenarios.

### 2.1 Task background and linguistic challenges

Word segmentation is a fundamental yet challenging task for the successful computational processing of Sanskrit texts. Sanskrit is underpinned by a well-developed oral tradition. It exhibits systematic euphonic changes in verbal forms during speech, preserved in writing as Sandhi (Goyal and Huet, 2016). Sandhi causes sound shifts at lexical edges and combines morphemes (Dave et al., 2021), thereby blurring lexical boundaries and treating the sequence as a single character run. Such changes can involve deletion, insertion, or replacement of phonemes at word boundaries (Ali et al., 2025a).

Classical Sanskrit grammar records approximately 281 Sandhi protocols (Reddy et al., 2018), which specify phonetic adjustment patterns that complicate the automatic splitting of words. Sandhi splitting supports several downstream tasks such as text classification (Sandhan et al., 2019; Krishna et al., 2016b), morphological tagging (Gupta et al., 2020), dependency parsing (Sandhan et al., 2021; Krishna et al., 2020), and automatic speech recognition (Kumar et al., 2022).

Although Sandhi formation is limited by deterministic phonological rules, the reverse process, splitting (viccheda), is more complex, being non-deterministic and extremely ambiguous (Aralikatte et al., 2018). The same fused expression can yield multiple acceptable segmentations; sometimes, a single fused expression has many splits. The correct disambiguation is thus based on semantic compatibility among candidate segmentations.

Previous research on compound (Sandhi) splitting in Sanskrit provides important insights into the mechanisms of word formation applicable to the Dravidian languages. Computational modeling of Sanskrit word formation may serve as a scalable model of morphological organization for other Indian languages from a natural language processing perspective (Bharati et al., 2006).

### 2.2 Word segmentation beyond Sanskrit

Segmentation of compound words is not confined to Sanskrit; similar phenomena occur in other languages, such as Thai (Haruechaiyasak et al., 2008), Chinese, and Japanese, which lack explicit word delimiters (Xue, 2003). To reduce feature engineering for Chinese language processing (Zheng et al., 2013; Pei et al., 2014; Yao and Huang, 2016), various sequence labeling methods have been proposed to assign labels to each character and split compound words. In Japanese, processes such as *rendaku*, a sequential voicing, result in phonological alternation in compounds, similar to Sandhi (Aralikatte et al., 2018). The terms *hana* (flower) and *hi* (fire) combine to form the compound word *hanabi*, in which the voiceless *h* of *hi* is voiced to *b*. Similar boundary transformations occur in Sanskrit Sandhi. For example, the combination *tat + api* becomes *tadapi*, where the final voiceless *t* turns into voiced *d* before a vowel. Nonetheless, Sanskrit presents one major challenge: not only must boundaries be found, but the model must also reconstruct phonologically altered characters at the split positions.

Chinese and Japanese do not exhibit Sandhi phenomena to the same extent as Sanskrit. The Chinese word segmentation proposed in Chen et al. (2015) is formulated as a character-level BMES sequence labeling task, and an LSTM-based segmenter aims to capture long-range dependencies by modeling them with gated memory. They model label dependence as a sentence-level structured inference layer, with learnable transition scores similar to Collobert et al. (2011) that decode the optimal overall BMES tag sequence. The model uses character embeddings, optionally including bigram embeddings and applies dropout for regularization. A neural network for Chinese word segmentation was proposed by Cai and Zhao (2016), in which, instead of assigning a tag to each character, the authors assign a score to each segmentation hypothesis. The model constructs word-candidate representations consisting of the individual characters that make them up using a gated combination neural network (GCNN) and an LSTM-based scoring model, thereby achieving coherence with the overall segmentation history via a language-model-style link score.

Sanskrit splitting is both a representation problem and a transformation recovery problem. The presence of these cross-linguistic similarities shows that phonological change at the morpheme boundary level is a general linguistic phenomenon. Identifying such correspondences may provide useful comparative insights and help improve segmentation models.

### 2.3 Sanskrit Sandhi splitting

In this subsection, we review prior work on Sanskrit Sandhi splitting, including rule-based, statistical, and neural approaches. Particular attention is given to how existing methods model boundary detection and segmentation, and to their limitations in capturing boundary uncertainty and handling multi-split scenarios.

#### 2.3.1 Rule-based, lexical, and classical methods

Most NLP systems for Sanskrit word splitting use both Panini's phonetic and morphological rules and lexical resources. Such systems are based on the application of formal methods (Huet, 2005; Goyal et al., 2007; Kulkarni and Shukl, 2009), but they may also rely on statistical methods, such as Dirichlet processes (Natarajan and Charniak, 2011), or finite-state methods (Mittal, 2010), graph-based approaches (Krishna et al., 2016a), or hybrid systems (Hellwig, 2015a). The main problem in Sanskrit word splitting is determining which of the possible splits of a compound word is most semantically correct. This challenge was addressed in Krishna et al. (2016a) by modeling word segmentation as a query-expansion task within a path-constrained random walk (PCRW) model. They also refined their model using morphological information via Inductive Logic Programming (ILP). This significantly improved performance.

Recent Sandhi splitting tools, including (i) the JNU splitter (Kumar, 2007), (ii) the INRIA Sanskrit Reader Companion (INRIA Sanskrit Dictionary, 2026), and (iii) the UoH splitter (Sanskrit Computational Linguistics, 2026), are mostly based on rule-based methods and have serious shortcomings because they are inflexible and are unable to deal with exceptions and variations. The work by Bhardwaj et al. (2018) on Sandhikosh provides an analysis and description of these tools and also proposes Sandhi and Sandhi Split data verification, a tool comparison, and work highlighting the limitations of rule-based approaches.

#### 2.3.2 Neural sequence labeling and sequence-to-sequence models

Neural approaches to Sanskrit Sandhi splitting have been explored through both sequence labeling and sequence-to-sequence formulations. Most earlier neural approaches are limited to single-split settings or rely on fixed-size windows, making them unsuitable for handling general multi-split scenarios. The study by Hellwig (2015b) models segmentation as a character-level sequence-labeling task, producing unsandhied outputs without relying on external linguistic resources. The algorithm is designed for shallow supervision, operating on gold-standard split transcripts and avoiding manually created grammatical pipelines or external lexical/morphological sources, making it well-suited to under-resourced settings. Similarly, Reddy et al. (2018) frames the problem as a sequence-to-sequence transduction task using encoder-decoder architectures, enabling direct generation of segmented forms. To address sparsity in a low-resource environment, they implement a vocabulary enhancement step using SentencePiece to learn subword-like units (GibberishVocab), train on and decode those units, and then map the predictions back to regular spacing for evaluation.

Dave et al. (2021) presents a sequence-to-sequence formulation that uses recurrent neural networks (RNNs) in a two-step algorithm. The model input is a compound word, and the output is its segmented form using a + symbol. An end-to-end neural model of Sanskrit word segmentation is proposed in Hellwig and Nehrdich (2018), which combines compound splitting and sandhi resolution, with tokenization being the task of turning an input sentence into a sequence of unsandhied tokens. Specifically, it considers a wide range of hybrid configurations that alternate the arrangement and interconnection of CNN and BiLSTM blocks (with shortcut connections), and optionally supplements character embeddings with simple n-gram-based split-probability cues based on training statistics and positions these models as a language-independent segmentation framework.

Subsequent work extends these approaches to jointly model boundary detection and generation. The Double Decoder RNN (DD-RNN) proposed by Aralikatte et al. (2018) introduces separate decoders for boundary prediction and sequence generation. Instead, Ali et al. (2025a) proposes a multi-task model for Sanskrit compound splitting that unifies split-location prediction and split generation within a single architecture, designed to minimize error propagation across the stages of a cascaded pipeline. The model operates at the character level and initially predicts a binary mask for a fixed-size Sandhi window region in which boundary transformations may occur, using two BiLSTM encoders (Hochreiter and Schmidhuber, 1997) (direct and reverse) fused with multi-head attention to capture co-occurring contextual information. A second branch performs sequence-to-sequence decoding to produce character segments as explicit boundary symbols and to model Sandhi-motivated character edits. However, these approaches are typically constrained to simplified settings or rely on local boundary windows, limiting their applicability to general multi-split scenarios.

More recent methods focus on boundary prediction, particularly in multi-split scenarios. ABBIE (Ali et al., 2025b) employs an attention-based bi-encoder architecture with parallel Bi-LSTMs, whose representations are combined via multi-head attention to predict split locations within a fixed Sandhi window. While effective for boundary detection, ABBIE does not generate the final segmented outputs. To address this limitation, Ali et al. (2026) proposes ABBIE-M, which extends ABBIE by formulating boundary prediction as a multi-label sequence task, producing a binary split indicator at each character position. It retains the attention-based bi-encoder architecture and uses an iterative thresholding algorithm to identify multiple split points without prior knowledge of their number. However, like ABBIE, it focuses solely on boundary prediction and does not perform sequence generation.

Overall, existing approaches rely on hard boundary predictions and fail to capture boundary uncertainty, thereby limiting robustness in ambiguous multi-split scenarios. They also depend on datasets that are often restricted to single-split compounds, limited in complexity, or not publicly available. Additionally, many neural models ignore structured dependencies between boundary labels, relying instead on independent or locally constrained predictions.

#### 2.3.3 Pretrained and transformer-based models

The authors of Nehrdich et al. (2024) describe ByT5-Sanskrit, a Transformer model pretrained at the byte level specifically for Sanskrit. They extend the token-free ByT5 architecture (Xue et al., 2022) by training the model on Sanskrit text, enabling it to take raw text as input without requiring external tokenizers or lexica. ByT5-Sanskrit is evaluated on established Sanskrit word-segmentation benchmarks. It outperforms prior data-driven methods, achieves accuracy comparable to the best lexicon-based models, and is more robust to out-of-vocabulary phenomena. However, ByT5-Sanskrit treats segmentation as a direct sequence generation task, without explicitly modeling boundary structure or uncertainty, which may limit its effectiveness in multi-split and highly ambiguous Sandhi cases.

TransLIST is a Sanskrit segmenter based on Transformers, presented in Sandhan et al. (2022), that combines the advantages of both lexicon-driven and knowledge-lean systems, using latent candidate information when available, while remaining usable without it. The LIST module of the model replaces the character sequence with span/word candidate nodes from the Sanskrit Heritage Reader (SHR), aligns them with the input using head-tail indices (and deterministic corrections for faulty matches), and falls back to n-gram spans when SHR candidates are missing. To utilize this hybrid representation more fully, TransLIST proposes soft-masked attention (SMA), in which self-attention is biased toward spans that contain the current character, and span-position encodings based on a relative head-to-tail distance feature are used. Predictions are generated by a classification head over an output vocabulary comprising short character sequences and an explicit terminator that defines segmentation. To correct invalid predictions, the model applies a PRCP path-ranking correction to the corrupted model output and enumerates SHR candidate paths to rescore them and identify invalid predictions. However, TransLIST relies on externally generated candidate spans and does not explicitly model boundary uncertainty or the interaction between boundary detection and generation, which are crucial in multi-split Sandhi scenarios.

Another recent study focused on the application of large language models for Sanskrit Sandhi splitting. In particular, Samarth and Mahalingam (2025) introduced The Gemma Sutras, a method that fine-tunes the Gemma-3 large language model for Sanskrit Sandhi splitting. This approach formulates the task as sequence generation and shows the ability of contemporary pretrained language models to learn Sanskrit Sandhi transformations and generate segmented output from compounds. While the experiment demonstrated the potential usefulness of applying large language models to the problem of Sanskrit morphological processing, in particular, learning the transformational rules from annotated examples, this method does not address other important aspects such as boundary localization and structured boundary label dependencies. Our approach, in turn, takes into account these important characteristics, as well as boundary probability and its uncertainty, which are calculated using CRF-based methods. Table 1 provides a comparative analysis on existing work of Sanskrit word segmentation approaches and models.

**Table 1:** Comparative analysis on existing work of Sanskrit word segmentation.

Method	Year	Architecture	BD	SG	UM	Key results^*^
(Hellwig 2015b)	2015	Char-RNN	✓	✓	×	Str.Acc. = **93.24%**
Reddy et al. (2018)	2018	Seq2Seq + Attn	×	✓	×	P/R/F1 = **90.77 / 90.30 / 90.53**
Hellwig and Nehrdich (2018)	2018	CNN + RNN	✓	✓	×	Sent/Str = **85.2% / 96.7%**
Aralikatte et al. (2018)	2018	DD-RNN	✓	✓	×	Loc/Sp = **95.0% / 79.5%**
Dave et al. (2021)	2021	2-stage Seq2Seq	✓	✓	×	Loc/Sp = **92.3% / 86.8%**
Sandhan et al. (2022)	2022	Transformer	Partial	✓	×	PM = **93.97% (S)**, **85.47% (H)**
Nehrdich et al. (2024)	2024	ByT5 Transformer	×	✓	×	PM = **90.11% (D)**, **93.83% (S)**, **94.29% (H)**
Samarth and Mahalingam (2025)	2025	Gemma-3 FT	×	✓	×	EM.Acc. = **87.7%**
Ali et al. (2025b)	2025	Bi-encoder	✓	×	×	Acc. = **89.27%**
Ali et al. (2026)	2026	Multi-label Bi-encoder	✓	×	×	Loc = **75.90%**; P_B_/R_B_/F1_B_ = **91.07% / 84.46% / 87.64%**
**Proposed model**	2026	Transformer + BiLSTM + CRF	✓	✓	✓	Loc/Sp/Seg = **84.39% / 79.34% / 87.79%**; P_B_/R_B_/F1_B_ = **92.84% / 91.66% / 92.25%**

^*^Reported scores are taken from the original papers and are not directly comparable across methods because the datasets and evaluation protocols differ. Bold values indicate the results of the proposed model.

BD, boundary detection; SG, segmentation generation; UM, uncertainty modeling; P/R/F1, precision/recall/F1-score; Sent, sentence accuracy; Str, string accuracy; Loc, location accuracy; Sp, split accuracy; Seg, segmentation accuracy; PM, perfect match; EM.Acc., exact-match accuracy; P_B_, boundary precision; R_B_, boundary recall; F1_B_, boundary F1-score. *Datasets:* D, DCS; S, SIGHUM; H, Hackathon.

In contrast to existing approaches, we propose a unified framework that jointly models boundary detection and sequence generation and explicitly leverages structured boundary information and uncertainty from CRF marginals to handle ambiguity in multi-split scenarios.

## 3 Problem formulation

The Sanskrit compound/Sandhi splitting is formulated as a joint structured prediction and conditional sequence generation problem. In particular, we formulate the problem in terms of two linked outputs that capture complementary aspects:

1. Boundary localization over the input word sequence, which identifies where a split should occur in the given compound;
2. Segmented output via conditional character generation, which produces the final segmented output.

Let the input compound be represented as a character sequence, as defined in Equation 1:


$$\begin{array}{c} X=\left(x_{1},x_{2},\ldots ,x_{L}\right),\;x_{i}\in V_{enc}, \end{array}$$ (1)

where *L* is the length of the input and 𝒱_*enc*_ is the encoder vocabulary.

The boundaries are encoded as a binary sequence, as defined in Equation 2:


$$\begin{array}{c} B=\left(b_{1},b_{2},\ldots ,b_{L}\right),\;b_{i}\in \left\{0,1\right\}, \end{array}$$ (2)

where *b*_*i*_ = 1 indicates that a split occurs after the character *x*_*i*_, and *b*_*i*_ = 0 indicates that no split occurs after *x*_*i*_. For example, *yasm*ā*jj*ā*yate* is a compound word, and its gold segmented form is *yasm*ā*t + j*ā*yate*. The corresponding boundary sequence is *B* = [0, 0, 0, 0, 0, 1, 0, 0, 0, 0, 0, 0], indicating a split between the sixth and seventh characters. This representation is defined directly over individual input characters and identifies split locations without incorporating output-side information into the boundary labels. In this formulation, we model the probability of a split at each position as *p*(*b*_*i*_ = 1|*X*), capturing boundary-related signals throughout the input sequence.

Furthermore, the problem involves generating a segmented character sequence from the input compound word, as defined in Equation 3:


$$\begin{array}{c} Y=\left(y_{1},y_{2},\ldots ,y_{T}\right),\;y_{t}\in V_{dec}, \end{array}$$ (3)

where 𝒱_*dec*_ is the decoder vocabulary that includes Sanskrit characters, the symbol +, and special tokens  < bos> (beginning of sequence) and  < eos> (end of sequence).

The output sequence includes explicit + separators and requires reconstructing any character-level changes introduced by Sandhi. In general, the segmented form is not obtained by simply inserting the + character into the input string because characters near split locations may undergo transformations or require restorations.

The segmentation process relies on both the probability of split positions and contextual information derived from the entire input sequence. The boundary sequence *B* indicates where splitting will occur, while the generated sequence *Y* defines the resulting segmentation, including both boundary placement and character-level modifications. For example, the compound word *yasm*ā*jj*ā*yate* is mapped to *yasm*ā*t + j*ā*yate*, where the original phonological forms must be recovered at the split boundaries, including Sandhi transformations such as the conversion of *jj* into *t + j*.

## 4 Proposed architecture

**Figure 1:**
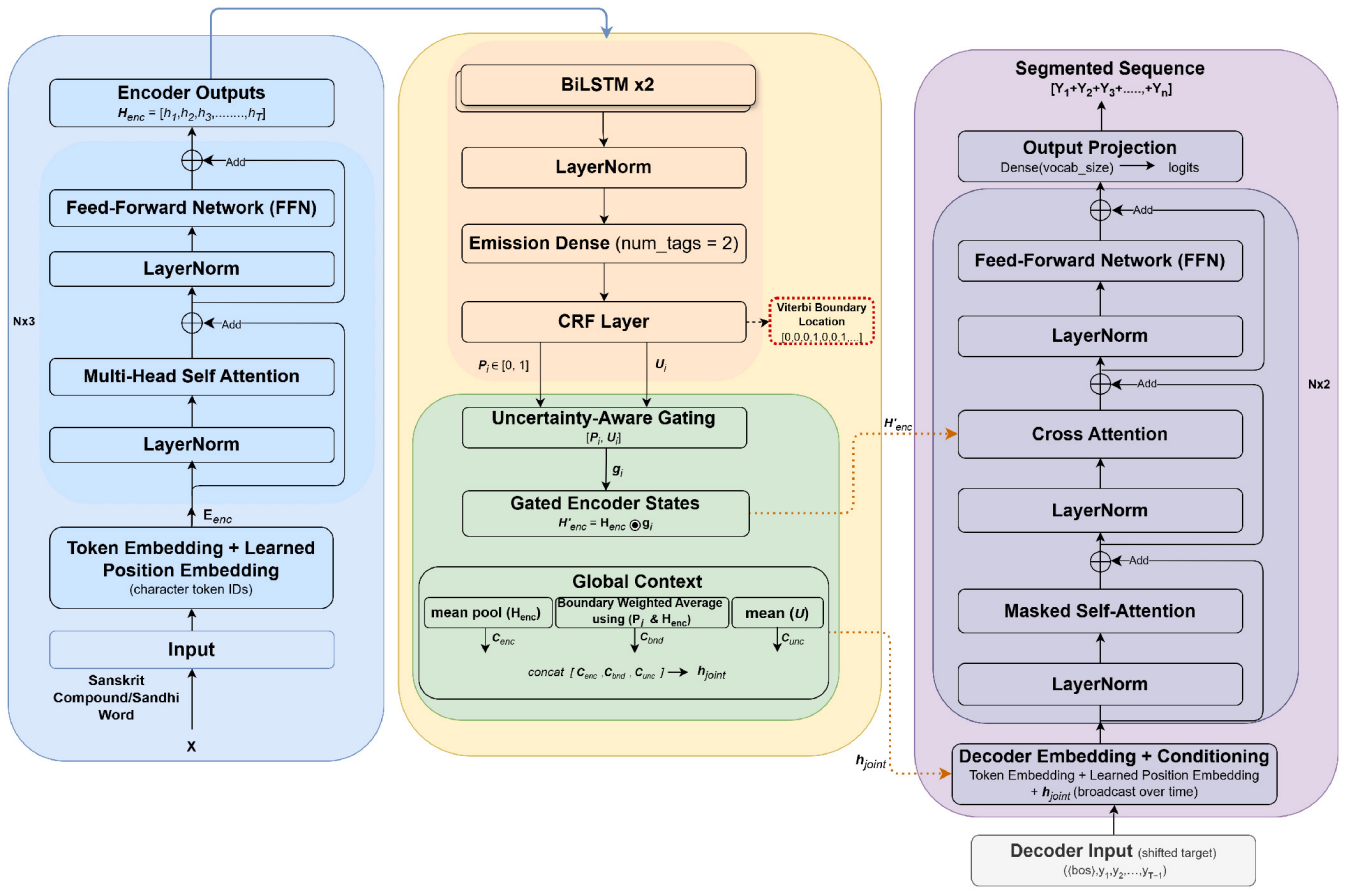
Proposed architecture for transformer encoder with BiLSTM-CRF boundary-head and boundary-aware transformer decoder.

The model illustrated in Figure 1 consists of a shared Transformer encoder, a stack of BiLSTM layers, a CRF-based boundary head, an uncertainty-aware gating unit, and a boundary-aware Transformer decoder. The proposed architecture is organized into three interrelated components as follows:

1. **Shared encoder:** a character-level Transformer that provides contextual representations *H*_enc_ for the input Sanskrit compound word.
2. **Boundary branch:** two stacked BiLSTMs followed by a linear-chain CRF that predicts structured boundary labels and produces posterior boundary marginals.
3. **Boundary-aware decoder:** a Transformer decoder that generates the segmented output while conditioning on (i) gated encoder states and (ii) a global boundary-aware context vector.

The encoder is shared between boundary localization and segmented sequence generation. The boundary branch is not merely an auxiliary predictor; its posterior boundary probabilities and uncertainty estimates are used explicitly to modulate encoder features and generate decoder conditioning signals. This design enables a fully joint model, rather than a rigid two-stage pipeline.

The architecture separates boundary localization from segmentation while linking them through posterior-based conditioning. This allows the model to leverage structured split-location evidence from the CRF branch without relying on hard boundary decisions, which is particularly useful when the Sandhi transformation affects the output side. Furthermore, the proposed architecture imposes no constraints on the number of boundaries in the input sequence. Unlike previous approaches, the proposed model can identify multiple split positions within a single compound word.

### 4.1 Shared transformer encoder

Each input character *x*_*i*_ is represented using a learned token embedding and a learned positional embedding as given in Equation 4.


$$\begin{array}{c} e_{i}=E_{\text{tok}}\left(x_{i}\right)+E_{\text{pos}}\left(i\right),\;e_{i}\in {\mathbb{R}}^{d}, \end{array}$$ (4)

and the embedded input sequence is defined in Equation 5


$$\begin{array}{c} H^{\left(0\right)}=\left[e_{1},e_{2},\ldots ,e_{L}\right]\in {\mathbb{R}}^{L\times d} \end{array}$$ (5)

where *L* represents the length of the input character sequence, and *d* denotes the dimension of the model. The encoder consists of *N*_*e*_ = 3 Transformer encoder layers as shown in Figure 1 following the self-attention architecture proposed by Vaswani et al. (2017), with LayerNorm (LN) (Ba et al., 2016). For ℓ = 1, …, *N*_*e*_, each layer applies pre-normalized multi-head self-attention (MHA) and feed-forward transformations (FFN), as defined in Equations 6 and 7:


$$\begin{array}{cc} {\tilde{H}}^{\left(\ell \right)} & =H^{\left(\ell -1\right)}+\text{MHA}\left(\text{LN}\left(H^{\left(\ell -1\right)}\right)\right), \end{array}$$ (6)

$$\begin{array}{cc} H^{\left(\ell \right)} & ={\tilde{H}}^{\left(\ell \right)}+\text{FFN}\left(\text{LN}\left({\tilde{H}}^{\left(\ell \right)}\right)\right). \end{array}$$ (7)

The final encoder outputs are defined in Equation 8


$$\begin{array}{c} H_{\text{enc}}=H^{\left(N_{e}\right)}=\left[h_{1},h_{2},\ldots ,h_{L}\right]\in {\mathbb{R}}^{L\times d}. \end{array}$$ (8)

The encoder produces a shared contextual representation that is used by both the boundary branch and the decoder. This shared representation improves parameter efficiency and enables the model to capture boundary-relevant features essential for segmented sequence generation.

### 4.2 Boundary prediction branch (BiLSTM–CRF)

The boundary branch formulates split detection as a structured sequence labeling problem over the encoder states *H*_enc_. It incorporates stacked BiLSTM layers for local sequential modeling, together with a CRF head for globally normalized boundary decoding. In Sanskrit compound words, boundary decisions primarily rely on short local character patterns occurring near Sandhi junctions. To capture explicit left-to-right and right-to-left sequential dynamics, the encoder states are passed through a two-layer bidirectional LSTM stack (Hochreiter and Schmidhuber, 1997), as defined in Equations 9–11:


$$\begin{array}{cc} Z^{\left(1\right)} & =\text{BiLST}{\text{M}}_{1}\left(H_{\text{enc}}\right), \end{array}$$ (9)

$$\begin{array}{cc} Z^{\left(2\right)} & =\text{BiLST}{\text{M}}_{2}\left(Z^{\left(1\right)}\right), \end{array}$$ (10)

where


$$\begin{array}{c} Z^{\left(2\right)}=\left[z_{1},z_{2},\ldots ,z_{L}\right],\;z_{i}\in {\mathbb{R}}^{d_{b}}. \end{array}$$ (11)

We set *d*_*b*_ = *d*_model_ by using *d*_model_/2 units per direction in each BiLSTM. This design complements the global context captured by the Transformer encoder with fine-grained local sequential modeling, which is crucial for accurate boundary detection. After normalization, a linear emission layer is applied to each *z*_*i*_ to produce scores for the two boundary labels, as defined in Equation 12:


$$\begin{array}{c} s_{i}=W_{e}z_{i}+b_{e}=\left[s_{i,0},s_{i,1}\right]\in {\mathbb{R}}^{2}, \end{array}$$ (12)

where *s*_*i, k*_ denotes the emission score for label *k*∈{0, 1} at position *i*, corresponding to *no-boundary* (*k* = 0) and *boundary* (*k* = 1).

#### 4.2.1 CRF for structured boundary decoding

To model the relationship between neighboring boundary labels, we apply a linear-chain Conditional Random Field (CRF) (Lafferty et al., 2001) on top of the emission scores. Let *A*∈ℝ^2 × 2^ denote the transition matrix, where *A*_*p, q*_ is the score of transitioning from label *p* to label *q*. For a candidate boundary sequence *B* = (*b*_1_, …, *b*_*L*_), the CRF score is defined in Equation 13 as


$$\begin{array}{c} Score\left(X,B\right)=\overset{L}{\underset{i=1}{\sum }}s_{i,b_{i}}+\overset{L}{\underset{i=2}{\sum }}A_{b_{i-1},b_{i}}. \end{array}$$ (13)

The conditional probability of *B* is then defined in Equation 14


$$\begin{array}{c} p\left(B|X\right)=\frac{\exp\left(\text{Score}\left(X,B\right)\right)}{\sum_{B^{\prime }}\exp\left(\text{Score}\left(X,B^{\prime }\right)\right)}. \end{array}$$ (14)

This formulation is well-suited for boundary detection because it models the entire boundary sequence jointly rather than making independent per-character label predictions. In Sanskrit compounds, valid split locations are sparse and localized, making independent predictions highly unstable. The CRF introduces sequence-level consistency by incorporating transition scores, enabling the model to capture local label dependencies while enforcing globally normalized predictions. The next section illustrates how these conditional probabilities are computed.

#### 4.2.2 CRF marginals and boundary uncertainty

In addition to the best boundary sequence (i.e., Viterbi decoding at inference), the model uses soft posterior marginals from the CRF to generate decoder-conditioning signals. Using the forward-backward dynamic program for linear-chain CRFs (Lafferty et al., 2001), We compute the unary marginals as defined in Equation 15


$$\begin{array}{c} m_{i,k}=p\left(b_{i}=k|X\right),\;k\in \left\{0,1\right\}. \end{array}$$ (15)

The forward and backward log-potentials are computed in Equations 16–19 as


$$\begin{array}{ccc} \alpha_{1}\left(k\right) & = & s_{1,k}, \end{array}$$ (16)

$$\begin{array}{ccc} \alpha_{i}\left(k\right) & = & s_{i,k}+\log\underset{k^{\prime }}{\sum }\exp\left(\alpha_{i-1}\left(k^{\prime }\right)+A_{k^{\prime },k}\right),\;i\geq 2, \end{array}$$ (17)

$$\begin{array}{ccc} \beta_{L}\left(k\right) & = & 0, \end{array}$$ (18)

$$\begin{array}{ccc} \beta_{i}\left(k\right) & = & \log\underset{k^{\prime }}{\sum }\exp\left(A_{k,k^{\prime }}+s_{i+1,k^{\prime }}+\beta_{i+1}\left(k^{\prime }\right)\right),\;i\leq L-1 \end{array}$$ (19)

and we define the normalization term *Z*(*X*) as the partition function in Equation 20


$$\begin{array}{c} \log Z\left(X\right)=\log\underset{k}{\sum }\exp\left(\alpha_{L}\left(k\right)\right). \end{array}$$ (20)

with the unary marginals defined in Equation 21


$$\begin{array}{c} m_{i,k}=\exp\left(\alpha_{i}\left(k\right)+\beta_{i}\left(k\right)-\log Z\left(X\right)\right). \end{array}$$ (21)

From the marginals, we define the boundary probability at position *i*:


$$\begin{array}{c} p_{i}=m_{i,1}. \end{array}$$ (22)

We also compute a per-position Shannon entropy as an uncertainty score:


$$u_{i}=-\sum_{k\in \{0,1\}}m_{i,k}\log\text{​}(m_{i,k}+\epsilon ),$$ (23)

where ϵ>0 is a small numerical constant. The sequence *p*_*i*_ encodes likely boundary locations, while *u*_*i*_ measures the uncertainty of boundary predictions at each input location. In particular, higher entropy indicates greater uncertainty, whereas lower entropy indicates more confident boundary predictions.

The CRF posterior marginals used for decoder conditioning are treated as decoupled signals, meaning gradients are not propagated through them when forming the conditioning signal. Both the boundary probability *p*_*i*_ and the uncertainty signal *u*_*i*_ are derived from the marginals and used to construct boundary-aware conditioning signals.

The BiLSTM-CRF branch provides both sequence-consistent boundary predictions and a calibrated posterior distribution over boundary locations, making it a suitable structured supervision signal and a source of probabilistic conditioning for the decoder. This formulation preserves boundary uncertainty at each position, which is particularly important for Sanskrit compounds, where multiple split configurations may be plausible.

### 4.3 Uncertainty-aware gating of encoder states

The decoder is expected to focus on encoder positions that are informative for splitting. To incorporate this information in a soft manner, we propose a position-wise gating mechanism based on boundary probabilities and uncertainties derived from CRF marginals. For each source position *i*, we define a boundary descriptor


$$\begin{array}{c} r_{i}=\left[p_{i};u_{i}\right]\in {\mathbb{R}}^{2}, \end{array}$$ (24)

and compute a scalar gate as defined in Equation 25


$$\begin{array}{c} g_{i}=\sigma \left(w_{g}^{⊤}r_{i}+b_{g}\right),\;g_{i}\in \left(0,1\right), \end{array}$$ (25)

where *w*_*g*_ and *b*_*g*_ are learnable parameters, and σ denotes the sigmoid function. The gating unit produces one scalar value per source location, which is broadcast across the hidden dimensions.

The encoder states are then modulated as defined in Equation 26:


$$\begin{array}{c} h_{i}^{\prime }=g_{i}h_{i}, \end{array}$$ (26)

yielding the gated encoder memory


$$\begin{array}{c} H_{\text{enc}}^{\prime }=\left[h_{1}^{\prime },h_{2}^{\prime },\ldots ,h_{L}^{\prime }\right]\in {\mathbb{R}}^{L\times d}. \end{array}$$ (27)

This gating mechanism preserves the full encoder sequence while adaptively reweighting it using boundary probability and uncertainty, enabling the decoder to attend to a boundary-aware representation without enforcing hard split decisions. Thus, this design mitigates error propagation from incorrect boundary predictions by treating boundary information as a soft, uncertainty-aware signal.

### 4.4 Global boundary-aware context construction

In addition to the local cross-attention over $H_{\text{enc}}^{\prime }$, the decoder is conditioned on a global vector that captures both the input and the outputs of the boundary branch. The global context is constructed from three complementary statistics, as illustrated in Figure 1.

We first compute token-level boundary probabilities *p*_*i*_ and uncertainties *u*_*i*_ (Equations 22, 23), and then aggregate encoder states into sequential-level summaries *c*_enc_, *c*_bnd_, and *c*_unc_ as defined in Equations 28–30.

*Mean encoder context:*


$$\begin{array}{c} c_{\text{enc}}=\frac{1}{L}\overset{L}{\underset{i=1}{\sum }}h_{i}\in {\mathbb{R}}^{d}, \end{array}$$ (28)

where *L* denotes the number of non-padding source positions. This vector captures the overall source-level context of the compound.

*Boundary-weighted encoder context:*


$$\begin{array}{c} c_{\text{bnd}}=\frac{\sum_{i=1}^{L}p_{i}h_{i}}{\epsilon +\sum_{i=1}^{L}p_{i}}\in {\mathbb{R}}^{d}. \end{array}$$ (29)

This representation emphasizes source positions that are likely split locations under the CRF marginals. *Mean boundary uncertainty:*


$$\begin{array}{c} c_{\text{unc}}=\frac{1}{L}\overset{L}{\underset{i=1}{\sum }}u_{i}\in \mathbb{R}. \end{array}$$ (30)

This scalar summarizes the overall uncertainty of boundary predictions.

All sequence-level summaries are computed over non-padding positions using the encoder padding mask. The three components are concatenated and projected to obtain a *joint global conditioning vector*, as defined in Equation 31:


$$\begin{array}{c} h_{\text{joint}}=\tanh\left(W_{joint}\left[c_{\text{enc}};c_{\text{bnd}};c_{\text{unc}}\right]+b_{joint}\right)\in {\mathbb{R}}^{d}, \end{array}$$ (31)

where $W_{joint}\in {\mathbb{R}}^{d\times \left(2d+1\right)}$ and $b_{joint}\in {\mathbb{R}}^{d}$. The resulting vector provides a compact global summary by combining (i) overall input context, (ii) boundary-focused information, and (iii) uncertainty estimates. This vector is broadcast to all decoder time steps.

The representations $H_{\text{enc}}^{\prime }$ (Equation 27) and *h*_joint_ provide complementary conditioning to the decoder: $H_{\text{enc}}^{\prime }$ captures boundary-aware local information via cross-attention, while *h*_joint_ provides global boundary-aware priors at each decoding step. This design treats boundary uncertainty as a probabilistic signal, enabling the model to emphasize confident boundary regions while downweighting ambiguous ones, and allowing the decoder to jointly exploit content, segmentation structure, and uncertainty.

### 4.5 Boundary-aware transformer decoder

The decoder generates the segmented output *Y* autoregressively, conditioning on both the gated encoder memory $H_{\text{enc}}^{\prime }$ and the global context *h*_joint_.

#### 4.5.1 Decoder input and global conditioning

During training, teacher forcing (Williams and Zipser, 1989) is used with the shifted decoder input sequence starting with  < bos>, while the decoder prediction target is the gold output sequence terminated with  < eos>, as defined in Equations 32 and 33


$$\begin{array}{c} Y^{\text{in}}=\left(\langle \text{bos}\rangle ,y_{1},y_{2},\ldots ,y_{T}\right) \end{array}$$ (32)

$$\begin{array}{c} Y^{\text{out}}=\left(y_{1},y_{2},\ldots ,y_{T},\langle \text{eos}\rangle \right) \end{array}$$ (33)

For decoder time step *t*, the input embedding is defined in Equation 34


$$\begin{array}{c} d_{t}^{\left(0\right)}=\sqrt{d_{\text{model}}}E_{\text{dec}}\left(y_{t}^{\text{in}}\right)+E_{\text{pos}}^{\text{dec}}\left(t\right)+h_{\text{joint}}. \end{array}$$ (34)

The token embeddings are scaled by $\sqrt{d_{\text{model}}}$ to maintain a consistent magnitude relative to the positional embeddings. The global context vector *h*_joint_ is broadcast across all decoding steps and added to the input embeddings, ensuring that each decoding step is informed by both local boundary-aware representations and global boundary context prior to attention.

#### 4.5.2 Decoder layers and cross-attention

The decoder includes *N*_*d*_ = 2 Transformer decoder layers, as shown in Figure 1 (Vaswani et al., 2017). Let $D^{\left(0\right)}=\left[d_{1}^{\left(0\right)},\ldots ,d_{T}^{\left(0\right)}\right]$. For ℓ = 1, …, *N*_*d*_, each layer applies masked self-attention, cross-attention to $H_{\text{enc}}^{\prime }$, and a feed-forward network are defined in Equations 35–37:


$$\begin{array}{cc} {\tilde{D}}^{\left(\ell \right)} & =D^{\left(\ell -1\right)}+\text{MH}{\text{A}}_{\text{self}}\left(\text{LN}\left(D^{\left(\ell -1\right)}\right)\right), \end{array}$$ (35)

$$\begin{array}{cc} {\hat{D}}^{\left(\ell \right)} & ={\tilde{D}}^{\left(\ell \right)}+\text{MH}{\text{A}}_{\text{cross}}\left(\text{LN}\left({\tilde{D}}^{\left(\ell \right)}\right),H_{\text{enc}}^{\prime }\right), \end{array}$$ (36)

$$\begin{array}{cc} D^{\left(\ell \right)} & ={\hat{D}}^{\left(\ell \right)}+\text{FFN}\left(\text{LN}\left({\hat{D}}^{\left(\ell \right)}\right)\right). \end{array}$$ (37)

Autoregressive decoding is enforced by masked self-attention, and alignment between the partially generated output and the boundary-informed source memory is achieved via cross-attention.

#### 4.5.3 Output projection

The final decoder states are projected onto the decoder vocabulary space as defined in Equation 38:


$$\begin{array}{c} o_{t}=W_{o}d_{t}^{\left(N_{d}\right)}+b_{o}, \end{array}$$ (38)

and the conditional next-token distribution is defined in Equation 39:


$$\begin{array}{c} p\left(y_{t}|y_{<t},X\right)=\text{softmax}\left(o_{t}\right). \end{array}$$ (39)

Since the decoder vocabulary includes Sanskrit characters and the separator “+,” the model directly generates the final segmented string, including Sandhi restorations. This decoder design enables both segmentation and Sandhi restoration to be performed within a single autoregressive process while explicitly accounting for structured boundary evidence and its uncertainty.

During inference, boundary labels are decoded using Viterbi from the CRF, and the segmented sequence is generated via greedy decoding, as defined in Equations 40 and 41:


$$\begin{array}{c} \hat{B}=\arg\underset{B}{\max}p\left(B|X\right), \end{array}$$ (40)

$$\begin{array}{c} {ŷ}_{t}=\arg\underset{y_{t}}{\max}p\left(y_{t}|{ŷ}_{<t},X\right), \end{array}$$ (41)

where decoding starts from  < bos> and continues until  < eos> is generated (or a maximum decoding length is reached).

## 5 Joint training objective

The model is trained end-to-end using a multi-task objective that combines boundary supervision with segmented sequence generation.

Given the gold boundary sequence B^*^, the boundary branch uses the CRF negative log-likelihood as defined in Equation 42:


$$\begin{array}{c} L_{\text{bnd}}=-\log p\left(B^{*}|X\right). \end{array}$$ (42)

In practice, the loss is normalized by the source length *L* to reduce length bias as defined in Equation 43:


$$\begin{array}{c} L_{\text{bnd}}^{\text{norm}}=-\frac{1}{L}\log p\left(B^{*}|X\right). \end{array}$$ (43)

Let $Y^{*}=\left(y_{1}^{*},\ldots ,y_{T}^{*}\right)$ be the gold segmented output and let $m_{t}^{\text{dec}}\in \left\{0,1\right\}$ be the decoder padding mask, where 1 indicates real tokens, and 0 indicates padding. The sequence generation loss is the masked token-level cross-entropy defined in Equation 44:


$$\begin{array}{c} L_{\text{seq}}=-\frac{1}{\sum_{t=1}^{T}m_{t}^{\text{dec}}}\overset{T}{\underset{t=1}{\sum }}m_{t}^{\text{dec}}\log p\left(y_{t}^{*}|y_{<t}^{*},X\right). \end{array}$$ (44)

The overall training objective is


$$\begin{array}{c} L=\lambda_{\text{bnd}}L_{\text{bnd}}^{\text{norm}}+\lambda_{\text{seq}}L_{\text{seq}}+\lambda_{\text{reg}}R\left(\Theta \right), \end{array}$$ (45)

where λ_bnd_ and λ_seq_ balance the boundary and sequence generation losses, and ℛ(Θ) denotes *L*_2_ regularization on the CRF transition parameters.

Joint optimization encourages the encoder to learn representations that are useful for both structured boundary prediction and segmented sequence generation.

## 6 Experimental results

The model is trained using mini-batch gradient descent with the Adam optimizer (Kinga and Adam, 2015) and gradient clipping. The initial learning rate is set to 5 × 10^−4^, and the batch size is 128. Training is performed for up to 60 epochs.

The model uses: three encoder layers, two decoder layers, *d*_model_ = 256, four attention heads, FFN size of 512, 128 BiLSTM hidden units per direction.

We apply dropout (Srivastava et al., 2014) at various stages to prevent overfitting. In addition to dropout and layer normalization, training uses two forms of explicit regularization. First, a decoupled weight decay of 10^−3^ is applied after each Adam update to selected parameters, excluding bias terms and normalization parameters, e.g., LayerNorm β, γ. Second, the CRF transition matrix is regularized with an *L*_2_ penalty of coefficient 10^−3^, which is added directly to the joint loss. The loss weights in Equation 45 are set to λ_bnd_ = 1.0 and λ_seq_ = 0.5.

We use early stopping based on validation location accuracy and train the model on an NVIDIA GeForce RTX 2070 GPU. The total number of parameters in our model is 4,173,629. Training of the proposed model took around 6.66 h. To further characterize the operational cost, we also evaluated the inference performance on the test dataset. The entire inference process took 130.64 s, yielding an inference latency of 14.57 ms per sample. Furthermore, we present an analysis of our model's complexity. Suppose that *L* denotes the length of the input sequence, *T* denotes the length of the output sequence, *d* denotes the dimension of the hidden representation vectors, and *K* denotes the number of boundary labels. Then, disregarding any constants and assuming a fixed number of Transformer layers and attention heads, the primary computational complexities of the model arise from the self-attention operations in the Transformer encoder and decoder, as well as encoder-decoder cross-attention. Thus, self-attention within the encoder layer takes *O*(*L*^2^*d*) time, self-attention in the decoder takes *O*(*T*^2^*d*) time, and cross-attention consumes *O*(*LTd*) time. The computation time required to perform the operations on the sequences using the BiLSTM component grows roughly linearly with *L*, and the decoding complexity of the CRF module is *O*(*LK*^2^). These additions provide a clearer understanding of the computational requirements of the proposed method and improve the reproducibility and practical assessment of the framework.

### 6.1 Dataset

We use the datasets described in Dave et al. (2021) and Aralikatte et al. (2018), both derived from the same source dataset, the UoH corpus (Sanskrit Computational Linguistics, 2026), which contains more than 100,000 examples. The SandhiKosh corpus (Bhardwaj et al., 2018) provides manually annotated splits. The research in Dave et al. (2021) considers only compound words with exactly two split parts, discarding multi-split cases. Conversely, Aralikatte et al. (2018) generalizes the setting by combining samples from both corpora.

In this study, we retain compound/sandhi words with varying numbers of splits, including multi-split compounds. We combine samples from both datasets to construct a comprehensive benchmark with diverse split structures. The dataset is preprocessed to remove duplicate entries, incorrect splits, samples with fewer than two segments, empty fields, and ambiguous compounds (i.e., identical compounds mapped to multiple gold outputs). We also remove length-mismatch outliers using a ratio threshold of 1.5 and an absolute difference threshold of 5.

Boundary indices are precomputed for each sample using a hybrid Sandhi-alignment procedure that combines global alignment and dynamic programming segmentation. The method selects the higher-confidence boundary assignment. The final dataset comprised 89,656 samples of varying lengths. The dataset is split into 80% training, 10% validation, and 10% test sets. The corpus is originally encoded in the International Alphabet of Sanskrit Transliteration (IAST) scheme and subsequently converted to the Sanskrit Library Phonetic (SLP1) (Learn Sanskrit, 2026) scheme, which is better suited for computational analysis due to its systematic phonetic representation.

### 6.2 Baseline comparison

Baseline experiments were selected for their task relevance and fairness in performance evaluation. The task at hand requires a word-level approach to Sanskrit compound splitting with explicit multi-split boundaries, whereas most recent segmentation models target sentence-level segmentation using different datasets or performance metrics, or tackle this task as an end-to-end sequence generation task with no explicit boundary predictions. Therefore, it would be unjustified to directly compare our results with these systems or to consider their metrics in any way during the experiments. For this reason, the choice of baseline models was limited to methods that also worked on the word-level task and produced outputs similar to ours.

We evaluate the performance of our approach against two baseline systems described below.

Unified Attention-Based Model (Ali et al., 2025a): This model proposes a character-level multi-task architecture for Sanskrit compound splitting that jointly performs split-location prediction and segmented sequence generation, thereby reducing error propagation. It first predicts the split location within a compound word using a BiLSTM encoder with multi-head attention, then generates a segmented output via a sequence-to-sequence decoder conditioned on the predicted boundary features. The original model is trained on a dataset containing only compounds with exactly two segments. We retrain the model in Ali et al. (2025a) on our dataset, which includes compounds with varying numbers of splits.

ABBIE-M (Ali et al., 2026): ABBIE-M focuses on Sanskrit compounds with multiple, unknown split locations through multi-label sequence prediction. It uses an attention-based bi-encoder with parallel BiLSTMs and iterative thresholding over a sliding window to identify high-confidence split locations without prior knowledge of the number of split positions. It does not generate the segmented outputs. We also retrain this model (Ali et al., 2026) on our benchmark.

Table 2 shows the results of the proposed model and two baseline systems on our benchmark dataset. The proposed model outperforms both baseline methods across boundary and splitting metrics. Compared with Ali et al. (2025a), our model improves Loc.Acc (84.39% vs. 77.32%), Sp.Acc (79.34% vs. 76.91%), and F1_B_ (92.25% vs. 85.45%). The model also improves over Ali et al. (2026) in Loc.Acc (84.39% vs. 75.90%), P_B_ (92.84% vs. 91.07%), R_B_ (91.66% vs. 84.46%), and F1_B_ (92.25% vs. 87.64%). These gains support the effectiveness of the proposed joint formulation, in which structured boundary prediction and segmented sequence generation are coupled through boundary-aware and uncertainty-aware decoder conditioning.

**Table 2:** Comparison with baseline systems on our benchmark.

Model	Loc.Acc.	Sp.Acc.	P_B_	R_B_	F1_B_
Ali et al. (2025a)	77.32	76.91	87.89	83.15	85.45
Ali et al. (2026)	75.90	-	91.07	84.46	87.64
Ours	**84.39**	**79.34**	**92.84**	**91.66**	**92.25**

Bold values indicate the best metric values.

We further compare our method against state-of-the-art approaches, including: (i) Rule-based systems (JNU, INRIA, UoH) (Kumar, 2007; INRIA Sanskrit Dictionary, 2026; Sanskrit Computational Linguistics, 2026), which are based on linguistic rules and lexical resources for Sanskrit Sandhi splitting. While effective in constrained settings, these systems rely on manually designed rules and are often inflexible, making them less robust to exceptions and variations. Their reported results are taken from Dave et al. (2021); Aralikatte et al. (2018). (ii) Double Decoder RNN (DD-RNN) (Aralikatte et al., 2018), which models Sanskrit compound splitting as a character-level sequence generation task. It employs a bidirectional LSTM encoder and two decoders: one for boundary prediction and one for generating segmented output, enabling joint modeling of split locations and Sandhi transformations. (iii) Sequence-to-sequence RNN model (Dave et al., 2021), which operates under a simplified setting restricted to compounds with a single split (two-part compounds), making it substantially easier than our benchmark. The model formulates Sandhi splitting as a sequence-to-sequence prediction task using recurrent neural networks.

### 6.3 Evaluation protocol and metrics

All models are evaluated on the test set. Table 3 summarizes the architectural differences. During inference, the boundary sequence is decoded using Viterbi decoding from the CRF, and the segmented output sequence is generated autoregressively with greedy decoding, starting from  < bos> until  < eos> is generated.

**Table 3:** Performance of the proposed model across different datasets.

Dataset	# Samples	Max. word length	Max # Splits	Loc.Acc	Sp.Acc	Seg.Acc	P_B_	R_B_	F1_B_
Dataset from Ali et al. (2026) (ours)	89808	124	9	92.21	89.14	96.12	95.95	95.62	95.79
Dataset from Dave et al. (2021) (ours)	77842	50	1	95.87	92.34	96.82	96.38	96.01	96.19
Dataset from Ali et al. (2025a) (ours)	100159	30	1	93.97	89.00	96.11	94.52	94.07	94.29
Our dataset (Ours)	89656	96	5	84.39	79.34	87.79	92.84	91.66	92.25

Let *N* be the number of test samples. For the *n*-th example, let ℬ_*n*_ and $\hat{ℬ}n$ denote the gold and predicted boundary-index sets, respectively, defined over valid boundary positions. Let **y**_*n*_ and ${\hat{\text{y}}}_{n}$ denote the gold and predicted segmented output token sequences. We use the following metrics.

Location accuracy: a word-level exact-match boundary metric. A sample is counted as correct only if the predicted boundary indices match the gold boundary indices as defined in Equation 46:


$$\begin{array}{c} Loc.Acc=\frac{1}{N}\overset{N}{\underset{n=1}{\sum }}\text{1}\left[{\hat{ℬ}}_{n}={ℬ}_{n}\right]. \end{array}$$ (46)

Splitting accuracy: a word-level exact-match segmentation metric. After greedy decoding, the predicted segmented sequence must match the gold sequence exactly as defined in Equation 47:


$$\begin{array}{c} Sp.Acc=\frac{1}{N}\overset{N}{\underset{n=1}{\sum }}\text{1}\left[{\hat{\text{y}}}_{n}={\text{y}}_{n}\right]. \end{array}$$ (47)

Segmentation accuracy: a character-level accuracy for the generated segmented output, not an exact-match metric. Let $Y^{\left(n\right)}=\left(y_{1}^{\left(n\right)},\ldots ,y_{T_{n}}^{\left(n\right)}\right)$ be the gold character sequence and ${Ŷ}^{\left(n\right)}=\left({ŷ}_{1}^{\left(n\right)},\ldots ,{ŷ}_{{\hat{T}}_{n}}^{\left(n\right)}\right)$ the predicted sequence. We compute a length penalized positional accuracy as defined in Equation 48:


$$\begin{array}{c} Seg.Acc=\frac{\sum_{n=1}^{N}\sum_{t=1}^{m_{n}}\text{1}\left[{ŷ}_{t}^{\left(n\right)}=y_{t}^{\left(n\right)}\right]}{\sum_{n=1}^{N}M_{n}},\\ m_{n}=\min\left(T_{n},{\hat{T}}_{n}\right),M_{n}=\max\left(T_{n},{\hat{T}}_{n}\right) \end{array}$$ (48)

where *T*_*n*_ and ${\hat{T}}_{n}$ represent the length of the gold and predicted segmented character sequences, respectively, for the *n*-th test sample. It measures partial correctness, even if the whole predicted split string is not exactly correct, correctly generated characters still contribute to the score, and it penalizes insertion/deletion errors via length mismatch.

Boundary (Precision/Recall/F1): they are computed as micro-averaged boundary metrics. Specifically, we accumulate global true positives (TP), false positives (FP), and false negatives (FN) across all valid boundary positions in all test examples, and then compute the *P*_*B*_, *R*_*B*_, and *F*1_*B*_, respectively.

### 6.4 Performance across different datasets

Table 3 reports performance of our model on three benchmark settings (Ali et al., 2026; Dave et al., 2021; Ali et al., 2025a) with different dataset sizes (# samples), maximum word lengths in characters (Max. Word length), and maximum numbers of split parts used in the compound word (Max # Splits). The results in Table 3 should be interpreted in light of dataset difficulty.

The setting in Dave et al. (2021) is restricted to compounds with a single split (two-part compounds), which is substantially simpler than our benchmark, where compounds may contain up to five split points (six-segment parts). Similarly, the benchmark settings vary in word length and split complexity.

In our benchmark, lower exact-match scores (Loc.Acc. and Sp.Acc.) are expected due to the increased difficulty of jointly identifying multiple boundary positions and generating Sandhi-restored segmented output. Despite this, the proposed model maintains a strong micro-averaged boundary performance.

Table 4 presents a comparison with state-of-the-art methods on the dataset from Dave et al. (2021). The results for JNU, UoH, and INRIA are described in Dave et al. (2021) and Aralikatte et al. (2018), which demonstrate the performance of rule-based tools on the test set. Our model improves Loc.Acc by 0.87 points and Sp.Acc by 12.84 points compared to Aralikatte et al. (2018). It also outperforms Dave et al. (2021) by 3.57 points in Loc.Acc and 5.54 points in Sp.Acc.

**Table 4:** Comparison with state-of-the-art methods on the dataset from Dave et al. (2021).

Model	Loc.Acc	Sp.Acc
JNU	-	8.1
UoH	-	47.2
INRIA	-	59.9
Aralikatte et al. (2018)	95.0	79.5
Dave et al. (2021)	92.3	86.8
Ours	**95.87**	**92.34**

Bold values indicate the best metric values.

**Figure 2:**
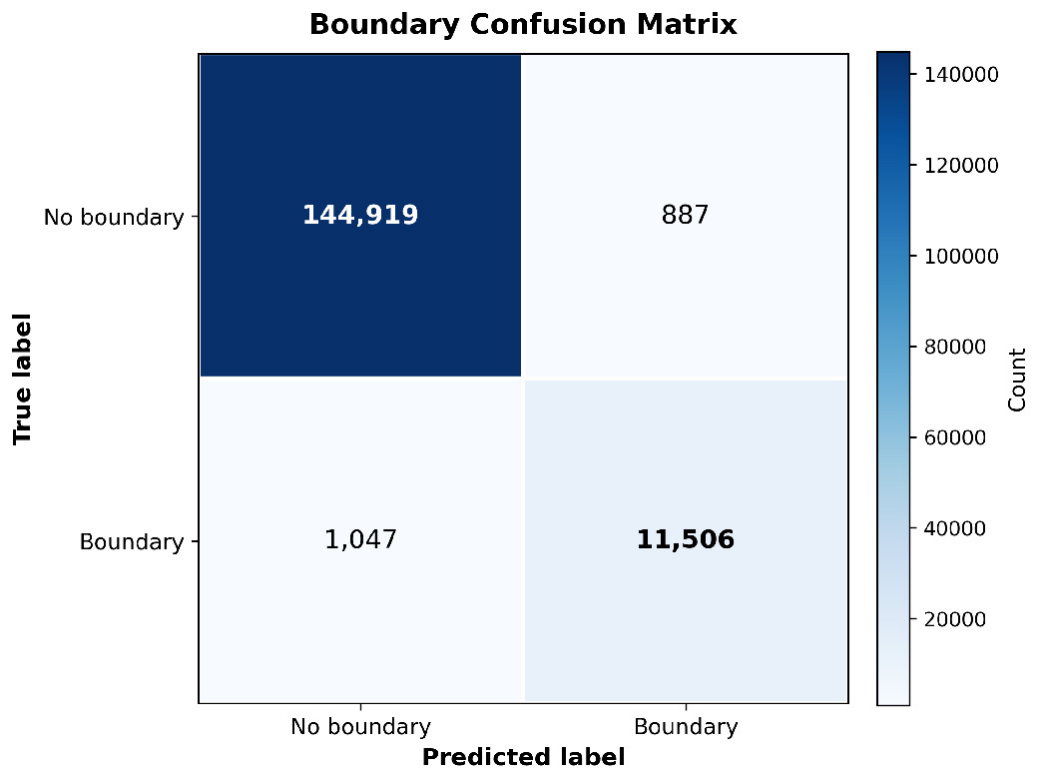
Boundary confusion matrix of the proposed method evaluated on our dataset.

Figure 2 shows the character-level confusion matrix for boundary Location prediction. Our model achieves strong boundary discrimination, correctly classifying most positions as either boundary or non-boundary. The low number of false positives and false negatives relative to true positives indicates high recall and precision in boundary detection.

**Figure 3:**
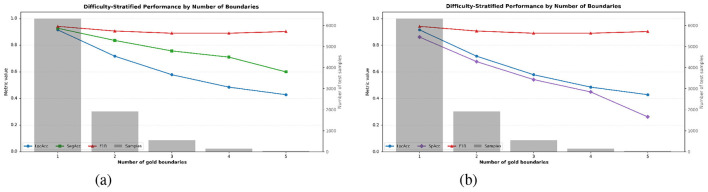
Difficulty-stratified performance analysis by number of boundaries. **(a)** LocAcc/SegAcc/F1_B_. **(b)** LocAcc/SpAcc/F1_B_.

Figure 3a shows that segmentation difficulty increases with the number of boundaries, affecting the exact match accuracies. As the number of boundaries increases, both Loc.Acc and Seg.Acc decreases, indicating that exact boundary recovery and character/token-level segmentation become more difficult for multi-split compound words. The gray bars in the figure further show that examples with a higher number of boundaries are less frequent. Despite this increased difficulty, the boundary-level F1_B_ score remains high across bins, suggesting that the model still identifies most split locations even in complex cases. The gap between exact boundary accuracy (Loc.Acc) and F1_B_ score suggests that most errors arise from partial misplacement, i.e., missing or extra split positions rather than failures in boundary detection and segmentation. Figure 3b reports the performance in terms of Loc.Acc, Sp.Acc and F1_B_. As the number of boundaries increases, both Loc.Acc and Sp.Acc decreases significantly, indicating the increasing difficulty of multi-split compound words. The stronger drop in Sp.Acc compared to Loc.Acc indicates that generating the exact segmented output is more challenging, even when split locations are partially correct. A small location prediction or decoding error can cause the entire segmented sequence to be counted as incorrect. Conversely, the F1_B_ score remains high across bins, indicating that the model continues to capture most of the boundary structure. This suggests that performance degradation in longer and more complex compounds is primarily due to stricter exact-match requirements, such as recovering the complete set of split locations and producing fully correct segmented outputs.

**Figure 4:**
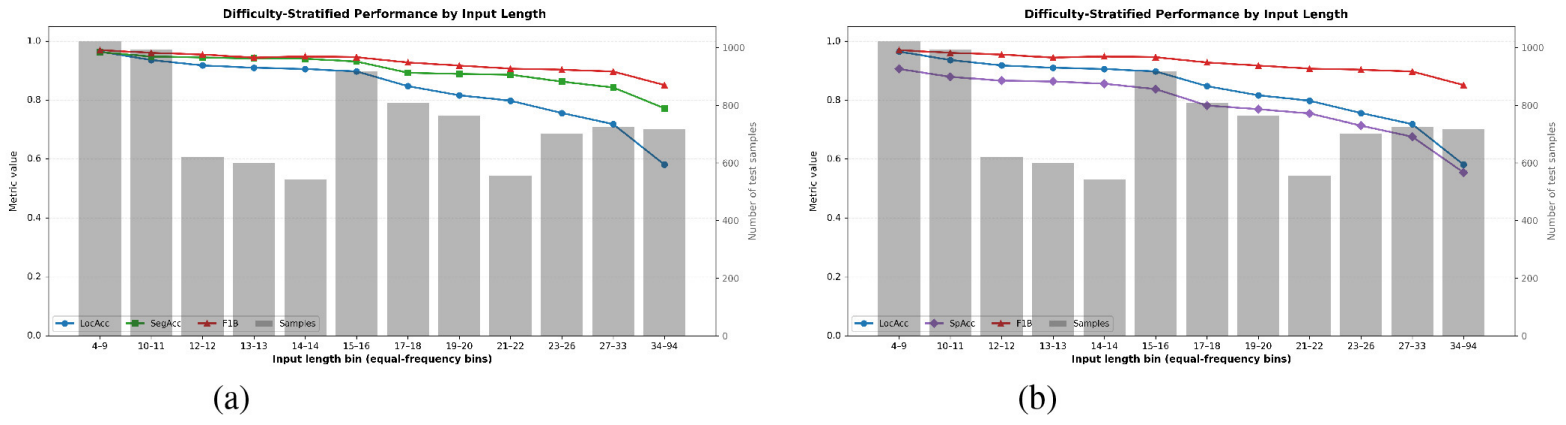
Difficulty-stratified performance analysis by input length. **(a)** LocAcc/SegAcc/F1_B_. **(b)** LocAcc/SpAcc/F1_B_.

Figure 4a groups test samples by compound length using equal-frequency bins, enabling a stable comparison between short and long compounds. The gray bars indicate the number of samples per bin. As the input length increases, both Loc.Acc and Seg.Acc decreases, indicating that exact boundary localization and character-level segmentation become more challenging for longer compound words. On the other hand, F1_B_ remains high and declines gradually. This indicates that the model continues to correctly identify more boundary locations, even when the segmentation is not entirely accurate. This suggests that errors in longer compounds are more often due to localized boundary misplacement or incomplete boundary sets, rather than to a complete failure of boundary detection.

Figure 4b reports the performance in terms of Loc.Acc, Sp.Acc, and F1_B_. As compound length increases, both Loc.Acc and Sp.Acc decreases, with a steeper drop in Sp.Acc. This means that reconstructing the exact segmented output is more challenging than identifying boundary locations. Even small boundary prediction or decoding errors can cause the entire segmented string to be counted as incorrect, despite many boundaries being correctly identified.

Meanwhile, F1_B_ is relatively stable and decreases more slowly, supporting the idea that the model has significant boundary-level accuracy with longer lengths. The increasing gap between Sp.Acc and F1_B_ with longer inputs indicate that, for long compounds, predictions are frequently locally accurate at some point but globally inaccurate under exact-match scoring, e.g., due to a single boundary error or a localized decoding error.

**Figure 5:**
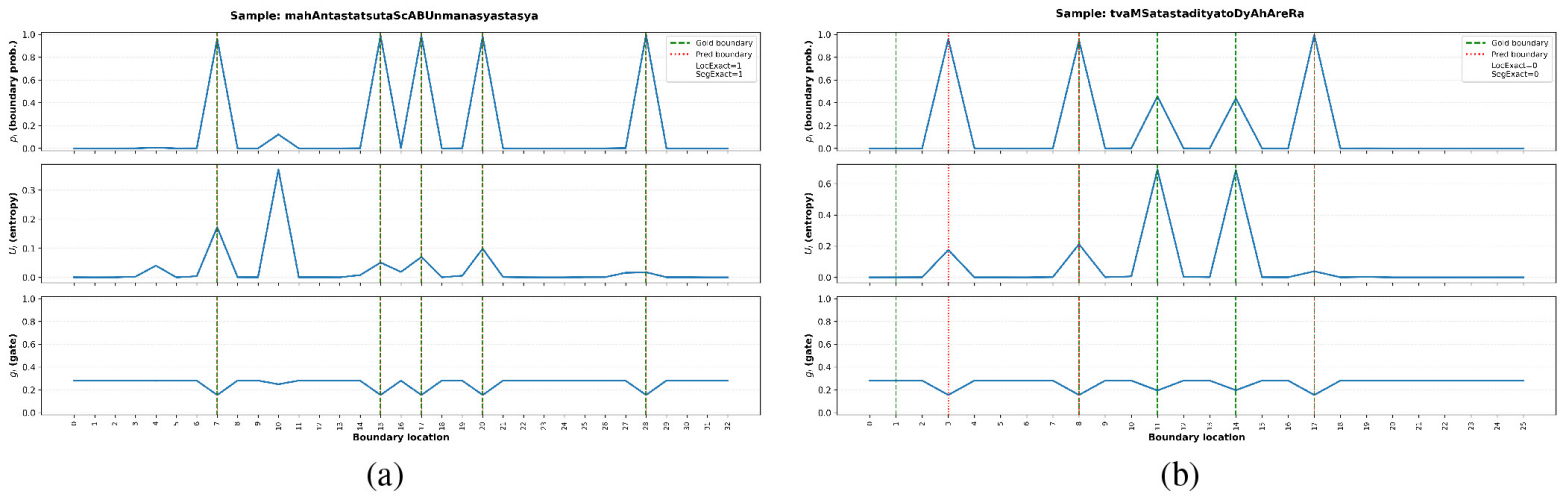
Uncertainty-aware gating for correct and incorrect predictions. **(a)** Correct prediction. **(b)** Incorrect prediction.

Figure 5a shows the token/character-level boundary probability *p*_*i*_, uncertainty *u*_*i*_ (entropy), and the gating *g*_*i*_ for the Sanskrit Sandhi word *mahAntastatsutaScABUnmanasyastasya*. The *x*-axis represents the boundary positions between characters, with position *i* marking the boundary between *x*_*i*_ and *x*_*i*_+1. Gold boundaries are shown as dashed vertical lines, while CRF-based Viterbi boundary predictions are shown as dotted vertical lines.

When the boundary predictions are correct, the CRF marginals show sharp peaks at the gold split positions, corresponding to high *p*_*i*_ and low entropy. This indicates a confident boundary decision, leading to correct boundary localization and segmentation (locExact=1 and SegExact=1). The entropy *u*_*i*_ is near zero almost everywhere and tends to increase around boundary-related decisions, reflecting localized uncertainty.

The gating values *g*_*i*_ remain stable overall but exhibit systematic modulation near boundary locations. Higher *g*_*i*_ values indicate that the corresponding encoder states are emphasized during cross-attention, whereas lower values suppress their contribution. This behavior demonstrates that the gating mechanism functions as a learned modulator, selectively highlighting informative boundary regions while attenuating less reliable regions. Lower gating values are expected near boundary peaks. Specifically, *g*_*i*_ is learned from [*p*_*i*_, *u*_*i*_] as a soft modulation signal for encoder states as mentioned in Equation 24, so it reflects decoder-side usefulness of a local representation, not simply boundary likelihood; As a result, a sharp CRF boundary peak does not require a corresponding peak in *g*_*i*_. This is particularly plausible in Sanskrit Sandhi, where the immediate neighborhood of the boundary often contains transformation-heavy characters, making the local source representation less reliable for direct cross-attention, while the boundary evidence is still retained through a separate global boundary-aware context.

Figure 5b shows a failure case for the Sanskrit compound *tvaMSatastadityatoDyAhAreRa*. In this example, the boundary probability *p*_*i*_ has multiple peaks that do not align with the gold boundaries. Although some split positions are correctly identified, others are incorrectly predicted.

In such cases, the CRF marginals *p*_*i*_ become more diffuse, and the entropy *u*_*i*_ increases, indicating higher uncertainty. The gating *g*_*i*_ exhibits weaker, less structured modulation, reducing its effectiveness at separating relevant regions. As a result, both boundary localization and segmentation fail (locExact=0 and SegExact=0).

**Bootstrap Confidence Intervals (CI):** To evaluate the stability of our reported results, we also computed 95% bootstrap confidence intervals on the held-out test set. The proposed model achieved 84.39% (95% CI: 83.66%–85.12%) location accuracy and 79.34% (95% CI: 78.51%–80.16%) segmentation exact-match accuracy with consistent performance when the evaluation data was resampled multiple times. The fairly narrow confidence intervals suggest that the results are quite reliable and robust to changes in the sample; that is, they are not dependent on any small subset of the test samples.

**Error Analysis:** To understand the behavior of the model, we conducted a detailed error analysis on the test set of 8, 965 instances. We predicted exact boundary positions for 7, 566 compounds. The remaining errors were 598 missing-splits, 457 extra-splits, and 344 mixed-splits. In particular, 622 of the boundary errors were near-miss predictions within one character position of the correct boundary, indicating that many failures stem from local boundary ambiguity. Regarding segmentation generation, 7, 113 outputs were exact matches, and the remaining errors were classified as 755 shorter-output, 611 longer-output, and 486 substitution cases. We also considered multi-split compounds, which are problematic because several boundary decisions may be correct. The model correctly predicted boundary locations in 1,775 of 2,640 multi-split examples and produced an exact segmentation in 1,664 cases. These results suggest that the main remaining issues are ambiguous boundary placement and complex multi-split compounds rather than failures of the sequence-generation component.

### 6.5 Ablation study

To analyze the contribution of each component in the proposed joint model, we conduct ablation studies. The encoder and decoder are kept fixed across all configurations; the ablations modify only the boundary branch and the global conditioning pathway, as described in Table 5. Table 6 reports the corresponding results.

**Table 5:** Proposed model configurations.

Model	Global context	BiLSTM	CRF	Sigmoid	Gating
Ours	✓	✓	✓	✗	✓
Ablation #1	✗	✓	✓	✗	✓
Ablation #2	✓	✗	✓	✗	✓
Ablation #3	✓	✓	✗	✓	✓
Ablation #4	✓	✓	✓	✗	✗

The encoder and decoder are present in all settings and are omitted for simplicity.

**Table 6:** Ablation results.

Model	Loc.Acc	Sp.Acc	Seg.Acc	P_B_	R_B_	F1_B_
Ablation #1	83.59	78.94	87.32	92.35	91.35	91.93
Ablation #2	82.21	78.24	86.86	91.62	90.90	91.26
Ablation #3	84.12	**79.44**	87.60	92.67	91.66	92.16
Ablation #4	84.23	78.50	86.87	92.47	**91.81**	92.14
Ours	**84.39**	79.34	**87.79**	**92.84**	91.66	**92.25**

Loc.Acc and Sp.Acc are exact-match metrics, Seg.Acc is character/token-level accuracy, and P_B_/R_B_/F1_B_ are micro-averaged boundary metrics. Bold values indicate the best metric values.

Removing global context (Ablation #1): removing the global context reduces the model's ability to inject sequence-level, boundary-aware information into the decoder. Compared with the full model, it reduces Loc.Acc from 84.39 to 83.59, Sp.Acc from 79.34 to 78.94, and F1_B_ from 92.25 to 91.93. These results indicate that the global context vector helps recover complete boundary structures and improves overall segmentation quality. This supports the effectiveness of the global context for strong decoding.

Effect of removing BiLSTM (Ablation #2): this configuration yields the lowest performance across almost all measures, including Loc.Acc (82.21), Sp.Acc (78.24), Seg.Acc (86.86), and F1_B_ (91.26). This suggests that the BiLSTM layers provide significant local sequential information before boundary prediction, thereby complementing the global context of the Transformer encoder. This kind of local modeling is important around Sandhi junctions, where boundary indications are often concentrated in short character spans.

Replacing CRF with a sigmoid head (Ablation #3): this variant shows mixed behavior. It slightly improves Sp.Acc from 79.34 to 79.44, while R_B_ remains unchanged. The full CRF-based model remains better on Loc.Acc (84.39 vs. 84.12) and Seg.Acc (87.79 vs. 87.60). The sigmoid head makes independent position-wise decisions, which can produce sharper local boundary activations and occasionally help exact segmented sequence generation. Conversely, the CRF jointly models label dependencies and enforces sequence-level consistency, which is more beneficial for strict exact boundary matching and stable structured prediction. More importantly, in our architecture, the boundary branch is not used solely for hard boundary labels: it also provides probabilistic boundary information, i.e., posteriors/marginals and uncertainty-aware conditioning for the decoder. The CRF-based variant is therefore more methodologically consistent with the proposed design, as it provides a structured, well-aligned boundary signal for both boundary prediction and confidence-aware sequence generation.

Effect of removing the gating mechanism (Ablation #4): we remove the gating mechanism for encoder states while keeping the CRF boundary branch and the global boundary-aware context unchanged. This ablation results in a modest yet consistent degradation in overall performance, with the most pronounced drops in the generation-oriented metrics, particularly in exact-match splitting (Sp.Acc) and character-level generation (Seg.Acc).

These results suggest that the gating mechanism mainly benefits the decoder-side generation rather than the boundary predictor. In our model architecture, boundary prediction is still produced by the BiLSTM-CRF branch. Conversely, gating is applied only after CRF marginals are computed and modulates the encoder representations that the decoder receives via cross-attention. Thus, removing gating has little effect on boundary detection but more clearly reduces segmentation quality, especially for exact-match splitting and character-level generation. It improves segmentation robustness under ambiguous boundary conditions.

### 6.6 Conclusion and future work

In this study, we presented a joint end-to-end Transformer-based multi-task architecture for boundary location prediction and segmentation of Sanskrit compound words. Our model uses a character-level Transformer encoder, which is shared between the BiLSTM-CRF and the Transformer decoder. The first branch, the BiLSTM-CRF, predicts the location within the Sanskrit compound word. The second branch, the Transformer decoder, is conditioned on the CRF marginal probabilities and entropy to construct gate-encoded states and boundary-aware global context, thereby enabling robust decoding under boundary ambiguity.

Experimental results on the benchmark clearly demonstrate that our model consistently outperforms existing state-of-the-art techniques, highlighting the benefits of coupled structured boundary prediction and sequence generation. While high-quality annotated data improves performance, the proposed architecture is specifically designed to handle complex multi-split scenarios and boundary ambiguity. Future work will focus on incorporating additional training data to improve performance and integrating linguistic knowledge into the model to better capture the contextual and semantic properties of Sanskrit compounds, including Sandhi phenomena. In particular, we will focus on studying more extensive, varied annotated corpora with larger samples of rare multi-splits. Moreover, we will consider applying data augmentation and semi-supervised learning techniques to address sparsity in less frequent cases. Additionally, future research will focus on techniques for uncertainty calibration and confidence-aware decoding to make the model more robust to boundary ambiguity. Finally, we will evaluate the proposed framework using additional Sanskrit corpora and related languages with rich morphology to assess its generalizability.

## Data Availability

The dataset used in this study is publicly available at: https://github.com/IrfanAliBabar/frontier_2026.
